# Early Thrombosis of Splenic Artery Stent Graft

**DOI:** 10.7759/cureus.16285

**Published:** 2021-07-09

**Authors:** Lalitha Padmanabha Vemireddy, Delaram Majlesi, Sonika Prasad, Nayha Tahir, Om Parkash, Hafiz Muhammad Jeelani, Maryna Shayuk

**Affiliations:** 1 Internal Medicine, Chicago Medical School, Chicago, USA; 2 Internal Medicine, Chicago Medical School, McHenry, USA; 3 Internal Medicine, Northwestern Medicine McHenry Hospital, McHenry, USA; 4 Internal Medicine, Chicago Medical School at Rosalind Franklin University of Medicine and Science, North Chicago, USA; 5 Internal Medicine, Chicago Medical School Internal Medicine Residency Program at Northwestern Medicine McHenry Hospital, McHenry, USA

**Keywords:** splenic artery aneurysm, splenic infarct, endovascular stent graft, stent graft thrombosis, dual anti-platelet therapy

## Abstract

Splenic artery aneurysms (SAAs) are among the most common visceral aneurysms behind aortic and iliac arteries. Certain factors like aneurysm size (especially giant SAAs), hypertension (HTN), symptomatology, pregnancy, portal hypertension (pHTN), and liver transplantation increase the risk of rupture. Most often found incidentally, but when symptomatic, can present with nonspecific symptoms like nausea, vomiting, anorexia, and epigastric/left upper quadrant pain. Diagnosis can be accomplished with different modalities of CT or MRI and digital subtraction angiography (DSA) being the gold standard for diagnosis. Treatment is usually preferred for aneurysms >2 cm, symptomatic cases, and pregnant women. Various surgical/interventional procedures can be performed and selected based on the patient’s sex, age, location of the aneurysm, size of the aneurysm, and presenting complaints/complications. Endovascular techniques with or without stent-graft placement are being used more, given the minimally invasive nature of these procedures. No clear guidelines exist on initiation of dual antiplatelet therapy (DAPT), but based on guidelines from visceral arterial stenting (especially iliac arteries and renal arteries), multiple case reports/series on SAAs, we highly recommend the usage of DAPT pre- and post-stent-graft placement to improve patency.

## Introduction

Splenic artery aneurysms (SAAs) are among the most common visceral artery aneurysms and are usually diagnosed incidentally, as close to 80% are asymptomatic [1-2]. The overall incidence of SAA based on retrospective studies is close to 0.8% and is more prevalent in females than males [1, 3]. Certain factors like aneurysm size >2 cm, symptomatic SAA, hypertension (HTN), pregnancy, portal hypertension (pHTN), and liver transplantation are associated with a higher degree of rupture and carry a high mortality rate if ruptured [1]. Modalities used in the treatment are open surgery, laparoscopic surgery, endovascular treatment (embolization and stenting), and medical treatment with close follow-up [4]. Endovascular treatment with coil embolization and stenting are frequently used, given the low surgical risk and morbidity. Based on the literature search, we now report a case of early splenic artery stent thrombosis extending into the celiac artery.

## Case presentation

A 42-year-old woman presented to the emergency department (ED) with complaints of palpitations and dyspnea. The patient complained of having palpitations daily with associated exertional dyspnea for three months prior to presentation. They used to last for about two minutes and subside on taking rest. For the last four months, she has been taking oral contraceptives for menorrhagia. There was no significant family history and she was a non-smoker, does not drink alcohol, and without any illicit drug abuse. Her vital signs upon arrival to the ED were stable. The cardio-respiratory examination was unremarkable. The initial electrocardiogram showed a normal sinus rhythm and axis, with no ST-segment changes.

**Figure 1:**
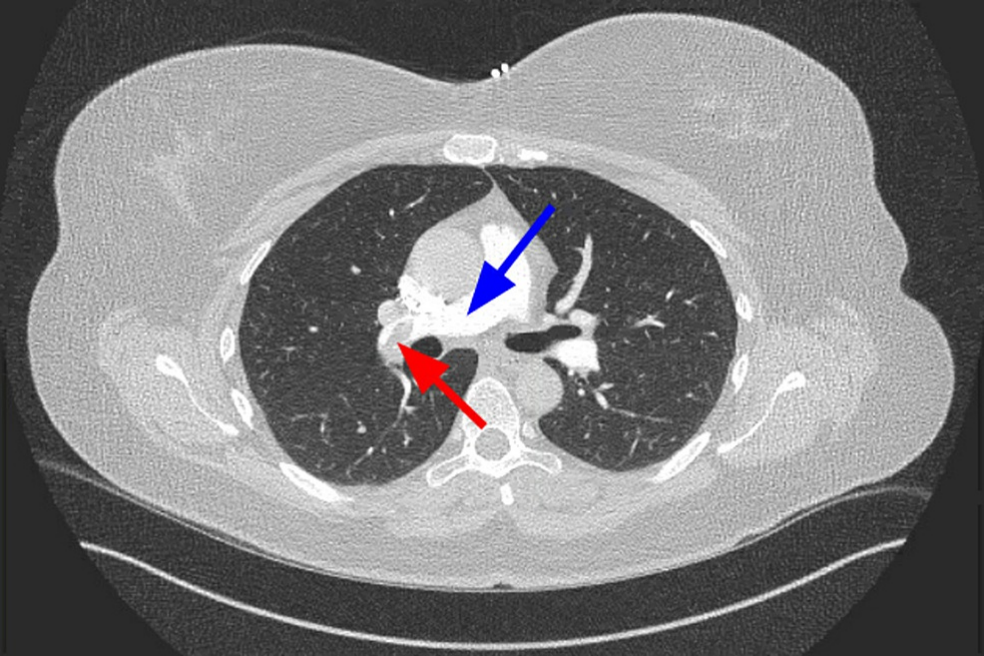
CTA PE protocol imaging showing right pulmonary artery (blue arrow) with thrombus in the distal right pulmonary artery (red arrow). CTA, computed tomography angiography; PE, pulmonary embolism

**Figure 2:**
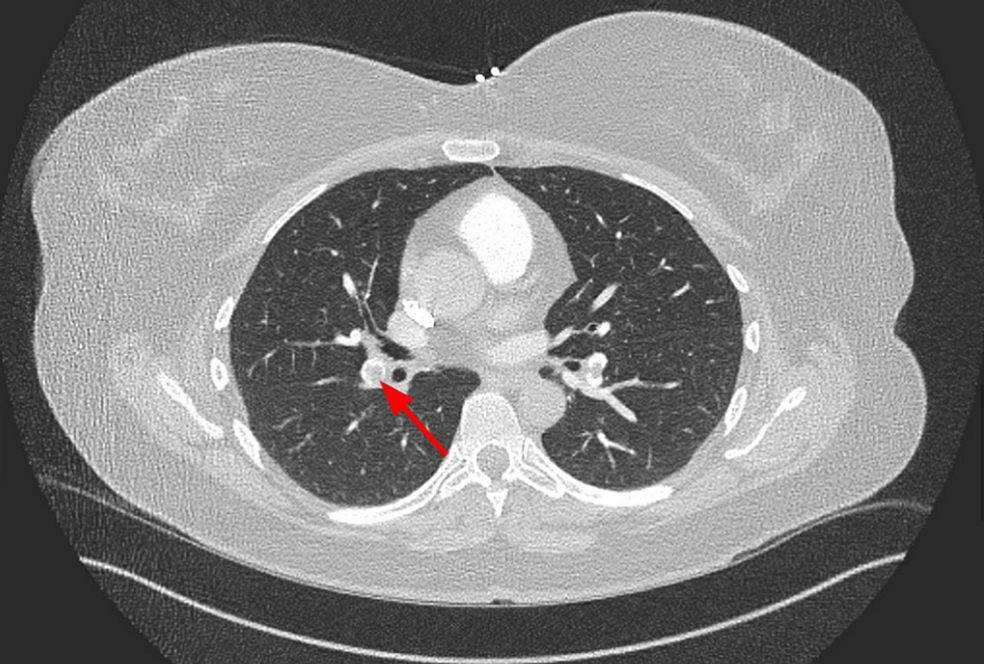
Redemonstration of thrombus in the right inter-lobar artery (red arrow) branching out from right pulmonary artery on CTA. CTA, computed tomography angiography

**Figure 3:**
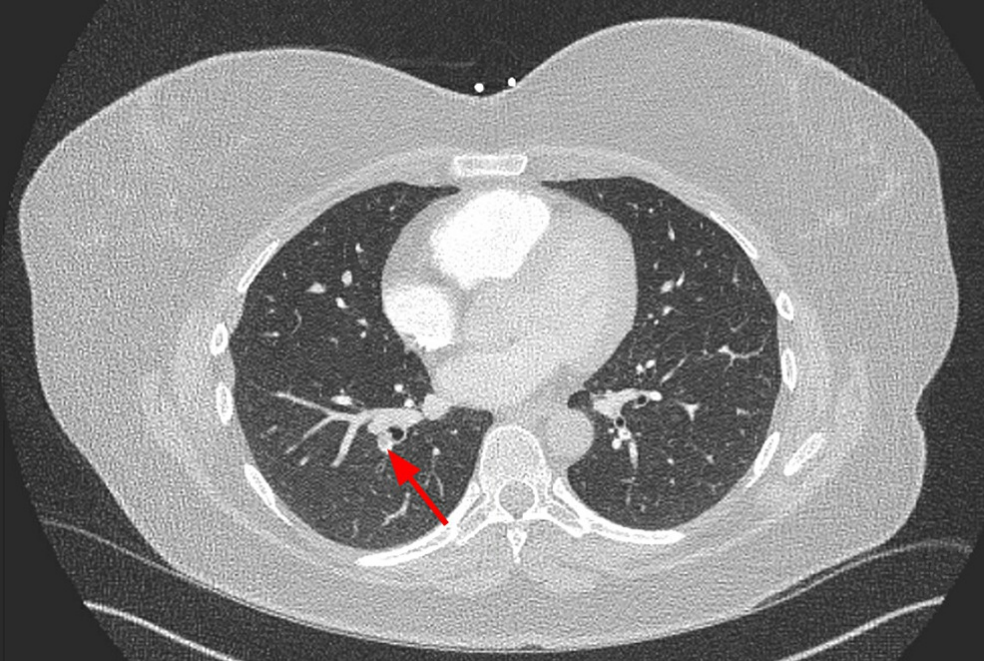
CTA showing thrombus in the segmental branch (red arrow) of the right inter-lobar artery. CTA, computed tomography angiography

CT angiography (CTA) of the chest demonstrated pulmonary emboli within the distal right main pulmonary artery, right upper lobar and right lower lobar/segmental branches, and left lower segmental branches (Figures 1-3). The D-dimer level was determined to be 1750 ng/mL. She had a 2D echocardiogram that did not show any increased pulmonary artery pressure or right heart strain. The lower extremity doppler showed no evidence of venous thrombosis. Subsequently, she was started on IV heparin infusion with close monitoring of the coagulation parameters.

**Figure 4:**
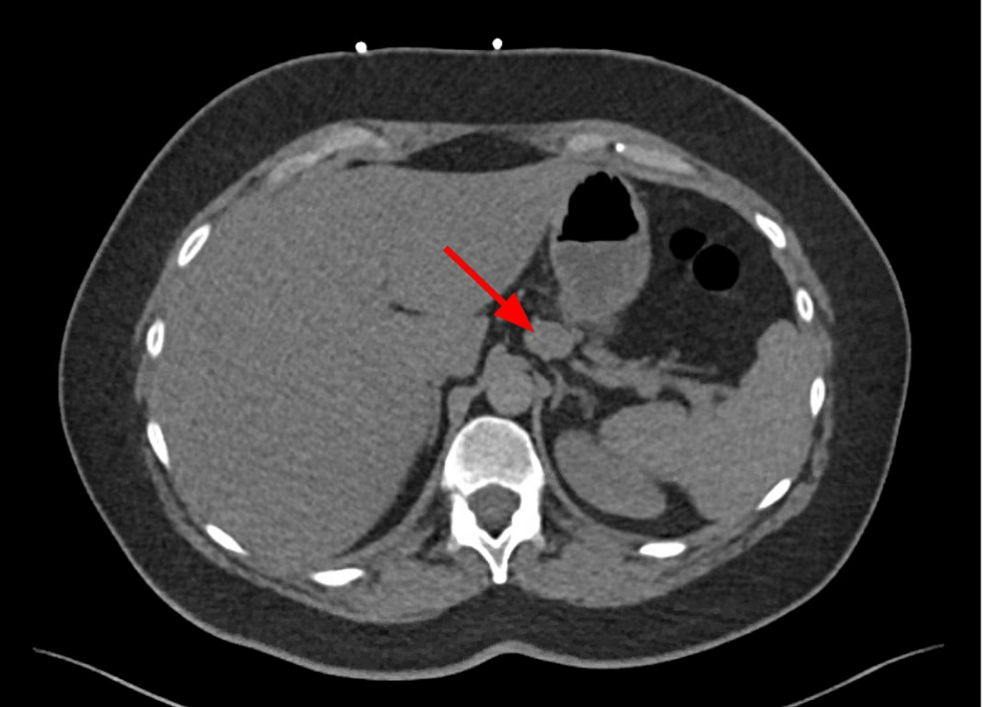
CTA showing a 2.2 cm exophytic lesion in the pancreas (red arrow); MRI recommended by the radiologist for further characterization of the lesion. CTA, computed tomography angiography

**Figure 5:**
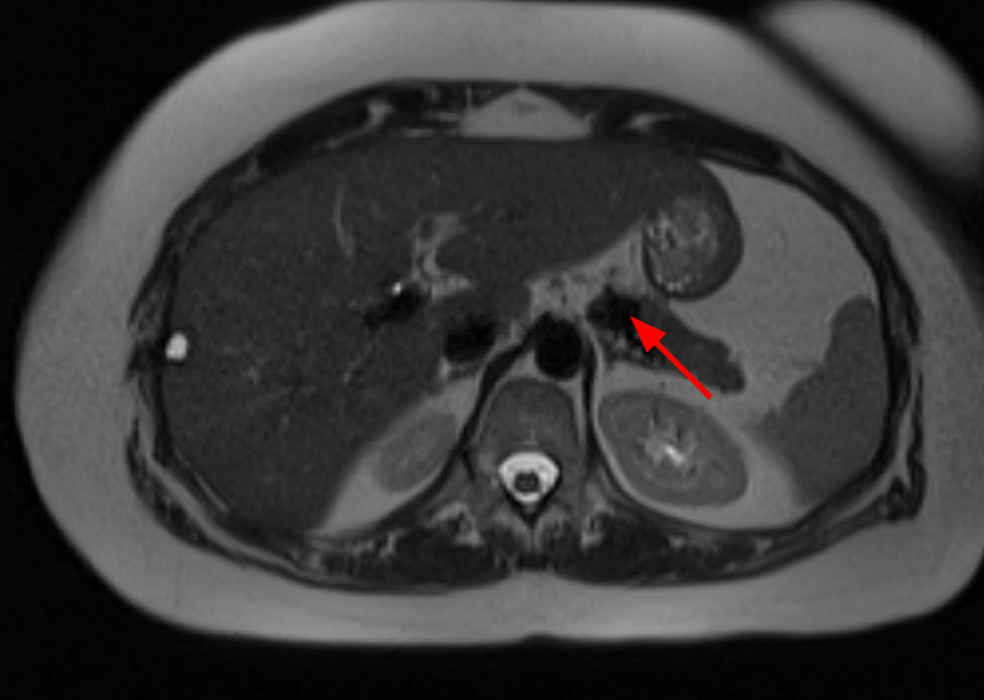
MRI showing a T2-hypointense lesion adjacent to the pancreatic body (red arrow), favored to represent an SAA. SAA, splenic artery aneurysm

A 2.2 cm exophytic pancreatic lesion was incidentally detected on CTA (Figure 4), and further imaging was recommended by radiology. The MRI of the abdomen demonstrated a homogeneously, T2-hypointense, T1-isointense structure, superior to the pancreatic body measuring 2.1 cm x 1.3 cm with delayed enhancement (Figure 5). Given the fact that the mass is T2 hypointense compared to normal pancreatic tissue and the adjacent location of the splenic artery, this was favored to be SAA. The radiologist also recommended a CTA abdomen and pelvis to delineate the aneurysm. No cystic or solid pancreatic lesions were observed.

**Figure 6:**
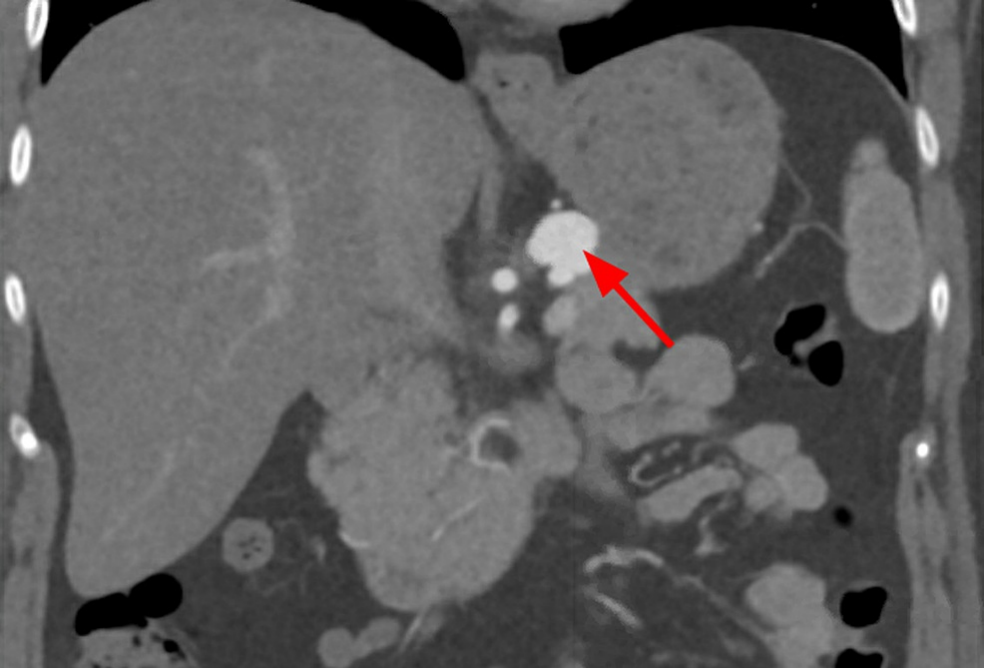
Coronal view of CTA abdomen and pelvis demonstrating the filling of contrast in the splenic artery due to an aneurysm (red arrow). CTA, computed tomography angiography

**Figure 7:**
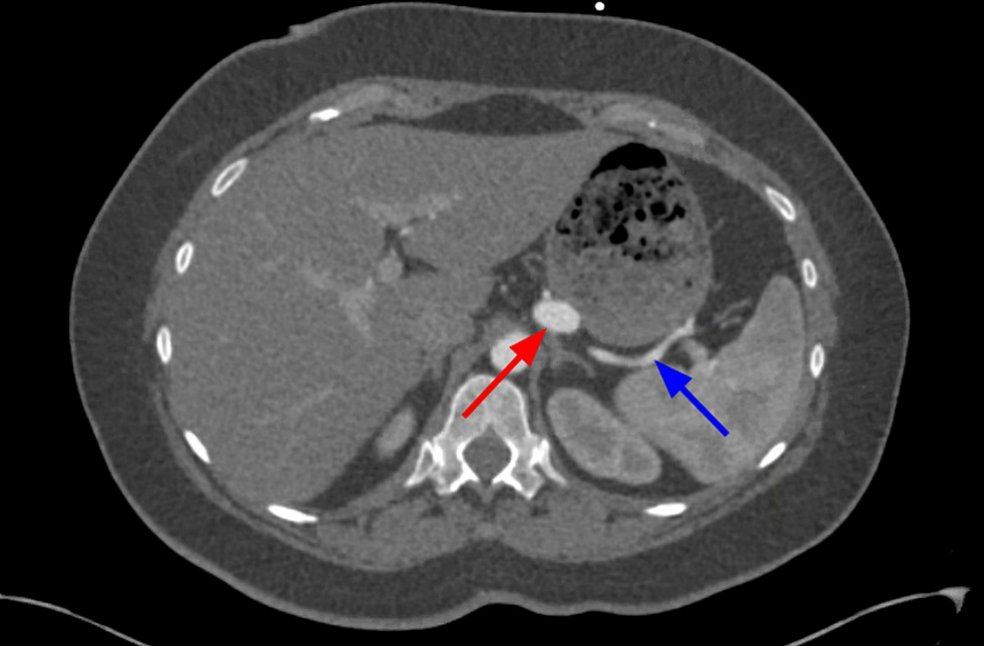
Axial view of CTA abdomen and pelvis demonstrating the splenic artery (blue arrow) with an aneurysm in the proximal part of the splenic artery (red arrow). CTA, computed tomography angiography

CT angiogram abdomen further demonstrated an SAA measuring 2.2 cm x 2.0 cm x 1.5 cm (Figures 6-7).

**Figure 8:**
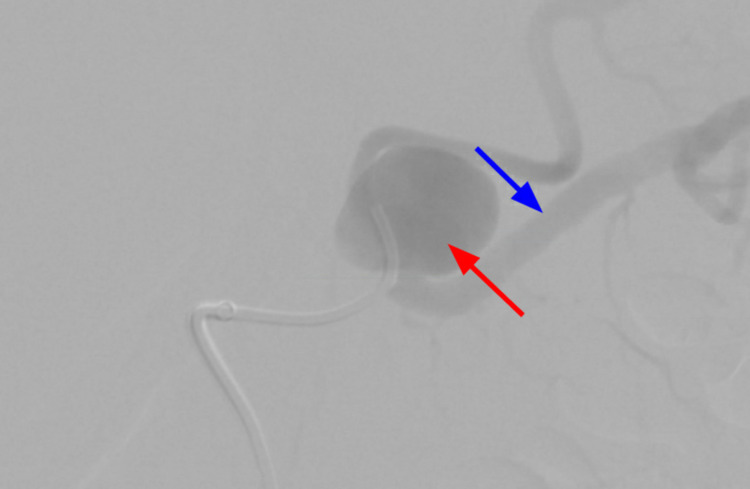
IR-guided splenic artery angiogram demonstrating the filling of contrast in the SAA (red arrow) and distal splenic artery (blue arrow). IR, interventional radiology; SAA, splenic artery aneurysm

**Figure 9:**
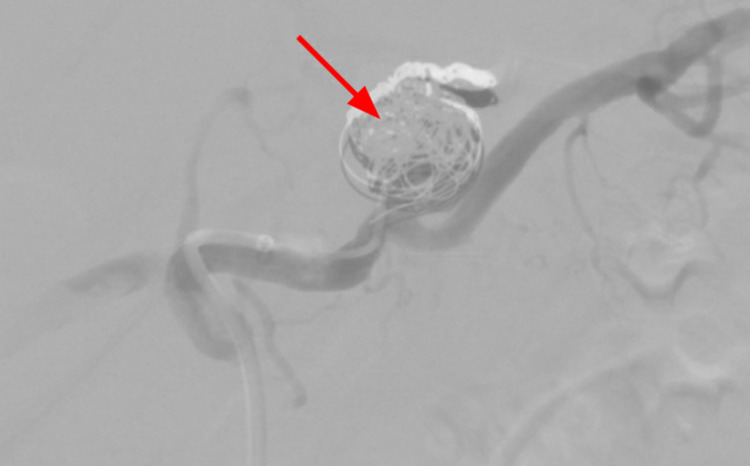
Coil embolization of the SAA (red arrow). SAA, splenic artery aneurysm

**Figure 10:**
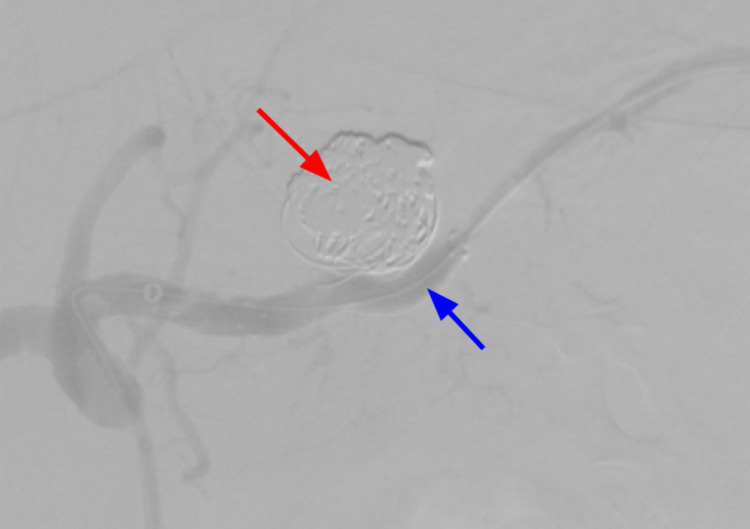
Deployment of the stent (blue arrow) and coil embolization (red arrow) with spasm noticed during the procedure distal to the stent.

Subsequently, interventional radiology performed coil embolization of the SAA (Figures 8-9), and a Viabahn 6 mm x 5 cm stent graft was deployed with mild spasm noticed distal to the stent during the procedure (Figure 10).

**Figure 11:**
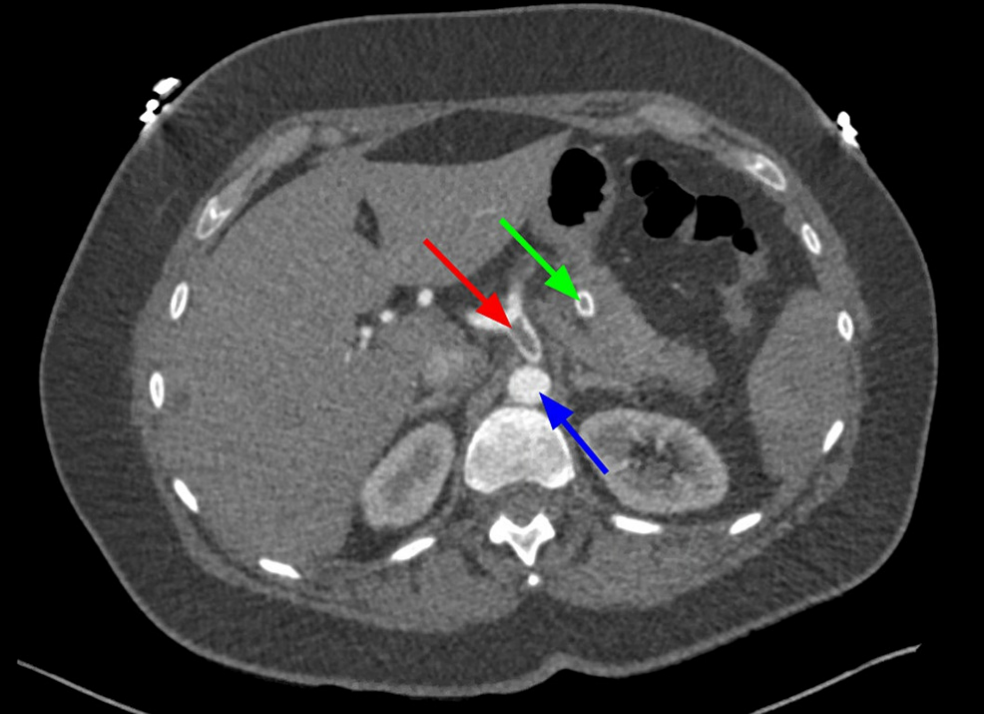
CTA abdomen and pelvis on POD#1 showing thrombus in the celiac artery (red arrow) right after bifurcation from descending aorta (blue arrow) and thrombus in the splenic artery (green arrow). CTA, computed tomography angiography; POD, post-operative day

**Figure 12:**
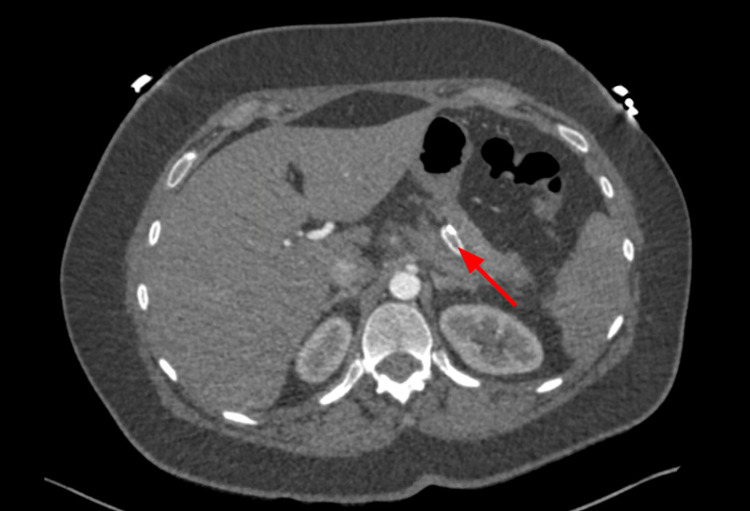
CTA abdomen and pelvis on POD#1 redemonstrating the thrombus in the splenic artery (red arrow). CTA, computed tomography angiography; POD, post-operative day

Heparin drip was discontinued two hours before the procedure. A few hours later, the patient developed left upper quadrant abdominal pain with gradual worsening. The following day she was found to have lactic acidosis (lactate level of 4.0 mMol/L) with elevated white blood cell count (WBC count of 21,000/𝜇L). CTA abdomen demonstrated a thrombus in the celiac axis and splenic artery with complete occlusion of the stent and distal splenic artery (Figures 11-12).

We initiated the patient on a heparin drip, 12 h after the initial apixaban dose. Repeat angiogram the following day demonstrated unchanged thrombus in the celiac trunk and splenic artery. The pain was typical for an infarcted spleen, and the interventional radiology consult team recommended no intervention. The patient had an elevation of lipase (618 units/L), which was likely due to thrombosis and ischemic pancreatitis. Lipase levels (332 units/L) and white blood cell count (21,000/𝜇L-17,100/𝜇L) at the time of discharge started improving.

**Figure 13:**
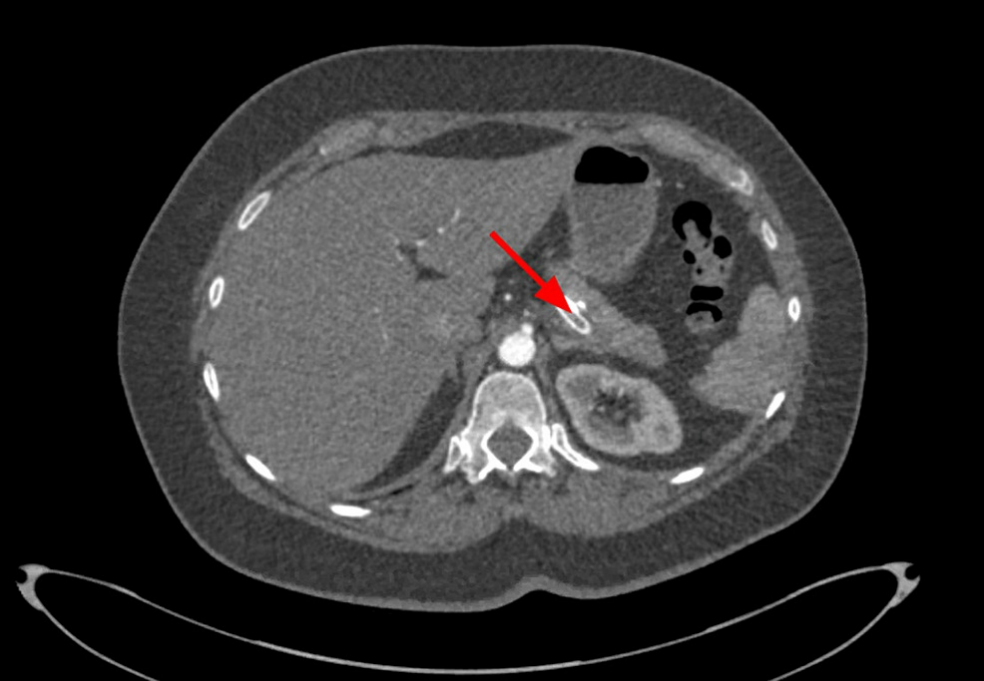
CTA abdomen and pelvis on POD#30 showing the splenic artery thrombosis (red arrow). CTA, computed tomography angiography; POD, post-operative day

**Figure 14:**
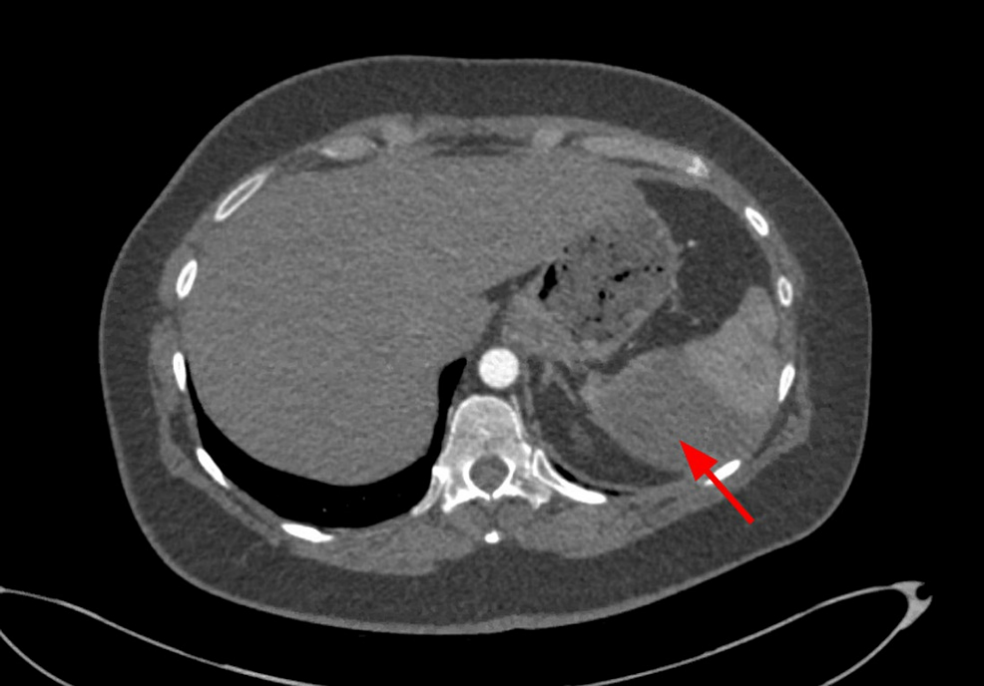
CTA abdomen and pelvis on POD#30 showing the infarct involving the spleen (red arrow) without any obvious abscess. CTA, computed tomography angiography; POD, post-operative day

An extensive workup to investigate the etiology of the pulmonary embolism and immediate splenic artery stent thrombosis was requested. Hypercoagulability testing panel including examination for proteins C and S, factor V Leiden, cardiolipin antibodies, antinuclear antibody (ANA) with reflex, rheumatoid factor, lupus anticoagulant was negative for any abnormalities. The patient got discharged from the hospital on oral anticoagulation and antibiotics. The follow-up imaging in one month redemonstrated evolving infarcts involving the spleen status post embolization of the splenic artery aneurysm (Figures 13-14).

## Discussion

Splenic artery aneurysms are defined as an abnormal dilatation of the splenic artery greater than 1 cm in diameter and greater than 10 cm in diameter described as giant SAAs [2]. SAAs are very commonly seen in women in their fifth and sixth decades of life [1, 5]. They are the third most common visceral aneurysms after aortic and iliac artery aneurysms [6]. HTN, hyperlipidemia (HLD), and tobacco smoking are the common comorbidities found in patients with SAAs, emphasizing atherosclerosis as an important factor in the causality of SAA [3]. Out of the factors mentioned earlier for increased risk of rupture, pHTN has been described to have very high mortality (54% in patients with pHTN vs 17% without pHTN) [1, 7]. In contrast to SAA, which involves the entire vessel wall, pseudoaneurysms (PAs) typically involve a focal point of the vessel; chronic pancreatitis and trauma have been described as the most important factors in the causation of PAs [8]. Unlike SAA, PAs are more common in men than women [2, 8].

Often, SAAs are found incidentally as close to 80% of the patients are asymptomatic [1-2, 9]. When symptomatic, the manifestations are nonspecific including nausea, vomiting, left upper quadrant/epigastric pain, and anorexia [2, 9]. Mattar and Lumsden found that up to 10% of patients with SAA have spontaneous rupture (higher predilection for giant SAA) with gastrointestinal bleeding, hemodynamic instability, sudden onset, sharp abdominal pain, and left shoulder pain (Kehr's sign) as presenting complaints [10]. In one series by Tessier et al., 58% of patients with PAs presented with hemodynamic instability as an initial presentation [8]. Lakin et al. also demonstrated that patients who presented with signs of rupture were more often to be <60 years of age. They also concluded that the amount of calcification was inversely proportional to the size of the aneurysm, but no definitive evidence was present if calcification was a protective factor against rupture or increasing size of the aneurysm [3]. 

Multiple studies can achieve the diagnosis of SAAs, including plain radiograph, ultrasound (US) doppler, contrast-enhanced CT (CECT), CTA, MRI, magnetic resonance angiography (MRA), multidetector CTA (MDCTA), and DSA [2, 4]. Plain radiograph and the US doppler (especially in pregnant patients) are inexpensive, non-invasive testing, which can help diagnose SAAs. Plain radiography is limited if the amount of calcification is not significant enough, and the US doppler can be influenced due to different factors like body habitus, aneurysmal size, and gas artifact [11-12]. CECT, CTA, MRI, and MRA can help provide quality images and should be chosen based on a case-by-case basis. MRI and MRA can be limited due to prosthetic metal devices/pacemakers, longer duration of the procedure, contrast exposure in chronic kidney disease (CKD), and unavailability. Contrast, radiation exposure, and limited usage in pregnancy could be potential restricting factors in using CECT, CTA, and MDCTA [4, 11]. DSA is the gold standard test for diagnosing SAA as it provides information about the exact location of the aneurysm, finding other aneurysms concomitantly, collateral circulation, and any potential rupture/bleeding source. It also has therapeutic benefits as we can perform coil embolization. The invasive nature of the procedure, arterial puncture, and complications post-arterial puncture are the main disadvantages of DSA [2, 4].

In terms of treatment, a conservative approach has been recommended with follow-up imaging every six months for patients with asymptomatic SAA <2 cm [2, 4, 9, 13]. Due to the increased risk of rupture, definitive treatment in symptomatic patients, asymptomatic SAA >2 cm, pregnancy, women of child-bearing age, patients undergoing liver transplantation, and portal hypertension (pHTN) patients is recommended [2, 4, 9]. All PAs require intervention as no relationship between the dimension of the aneurysm and rupture risk has been found [14]. Multiple factors including sex, age, location of the aneurysm, size of the aneurysm, and presenting complaints/complications need consideration when choosing an intervention. Open surgery remains the gold standard technique, but laparoscopic surgery and endovascular treatment with coil embolization/stenting are increasingly used [2, 4, 13]. Tortuosity of the splenic artery, location of the aneurysm, and decrease in arterial dimensions limit embolization and stent grafting [14]. The most common complications of coil embolization include splenic abscess, infarction, aneurysmal rupture, coil migration, and recanalization of the SAA [14,15]. Ouchi et al. performed a literature review on 17 cases who underwent endovascular stent grafting and the results showed a success rate of 100%, graft patency rate of 94%, reintervention rate of 0%, and splenic infarct rate of 11% [16]. Multiple case reports and series were published with the placement of stent graft for SAA using dual antiplatelet therapy (DAPT) pre-and post-operatively regardless of their coagulable state [13,17-18]. We believe that not initiating our patient on DAPT could have contributed to an SAA. To prevent stent thrombosis or restenosis, every individual should be initiated on DAPT pre-and post-operatively; however, the duration of DAPT post-operatively is controversial and varies from one to six months. DAPT is recommended to be initiated at least five to seven days before the procedure [19-22]. Based on our experience and literature review, we believe that DAPT pre- and post-stent-graft placement could improve the patency of the stent.

## Conclusions

Splenic artery aneurysms are a rare entity and most often found incidentally. Certain factors are associated with an increased risk in the rupture of SAAs. SAAs >2 cm requires intervention despite being asymptomatic. Open/laparoscopic surgery and coil embolization with or without stent grafting are treatment options and should be selected based on the case. Based on the case reports and literature review, we strongly recommend DAPT before and after stent-graft placement, at least until further evidence is available.
